# The Integral Role of Magnesium in Muscle Integrity and Aging: A Comprehensive Review

**DOI:** 10.3390/nu15245127

**Published:** 2023-12-16

**Authors:** Ana Carolina Remondi Souza, Andrea Rodrigues Vasconcelos, Denise Deo Dias, Geovana Komoni, José João Name

**Affiliations:** Kilyos Assessoria, Cursos e Palestras, São Paulo 01311-100, Brazil; anacarolina.souza@kilyos.com.br (A.C.R.S.); andrea.vasconcelos@kilyos.com.br (A.R.V.); denise.dias@kilyos.com.br (D.D.D.); geovana.komoni@kilyos.com.br (G.K.)

**Keywords:** magnesium, aging, sarcopenia, frailty, intrinsic capacity

## Abstract

Aging is characterized by significant physiological changes, with the degree of decline varying significantly among individuals. The preservation of intrinsic capacity over the course of an individual’s lifespan is fundamental for healthy aging. Locomotion, which entails the capacity for independent movement, is intricately connected with various dimensions of human life, including cognition, vitality, sensory perception, and psychological well-being. Notably, skeletal muscle functions as a pivotal nexus within this intricate framework. Any perturbation in its functionality can manifest as compromised physical performance and an elevated susceptibility to frailty. Magnesium is an essential mineral that plays a central role in approximately 800 biochemical reactions within the human body. Its distinctive physical and chemical attributes render it an indispensable stabilizing factor in the orchestration of diverse cellular reactions and organelle functions, thereby rendering it irreplaceable in processes directly impacting muscle health. This narrative review offers a comprehensive exploration of the pivotal role played by magnesium in maintaining skeletal muscle integrity, emphasizing the critical importance of maintaining optimal magnesium levels for promoting healthy aging.

## 1. Introduction

Aging is an inherent process characterized by significant physiological changes, encompassing a gradual reduction in cardiac output, increased blood pressure, diminished vital capacity, elevated blood glucose levels, cerebral tissue atrophy, neurotransmitter modifications, decreased bone and muscle mass, declining skin elasticity and tonicity, and compromised sensory functions, including those of vision, hearing, and taste [1,2,3]. The extent of physiological decline associated with aging demonstrates considerable interindividual variability, playing a fundamental role in an individual’s responsiveness to both daily and acute stressors, with these variations clinically being indicative of susceptibility and frailty [4].

Within a series of publications focusing on healthy aging, the World Health Organization (WHO) introduced the concept of intrinsic capacity (IC) [4,5,6]. IC encompasses five domains: locomotor, sensory, vitality, cognitive, and psychological, which correspond to an individual’s physical and mental capabilities [4,5,6,7]. The optimization and preservation of IC are pivotal for preventing frailty and fostering healthy aging.

An individual’s IC is contingent upon and susceptible to a range of elements that can be categorized as nonmodifiable factors (e.g., sex, age, and genetic factors) and modifiable factors (such as smoking, alcohol consumption, and diet). While the relationship between nutrition and aging is well established in the literature [8,9,10,11,12], most studies have primarily focused on how different dietary patterns may influence the prevention of diseases such as type 2 diabetes, cardiovascular disease, cancer, and Alzheimer’s disease [13,14,15,16,17,18,19]. Little attention has been given to the significance and role of nutrition, particularly micronutrients, in maintaining physiological homeostasis, which undoubtedly exerts a direct influence on IC maintenance and, consequently, on the reduction of frailty risk.

**Figure 1:**
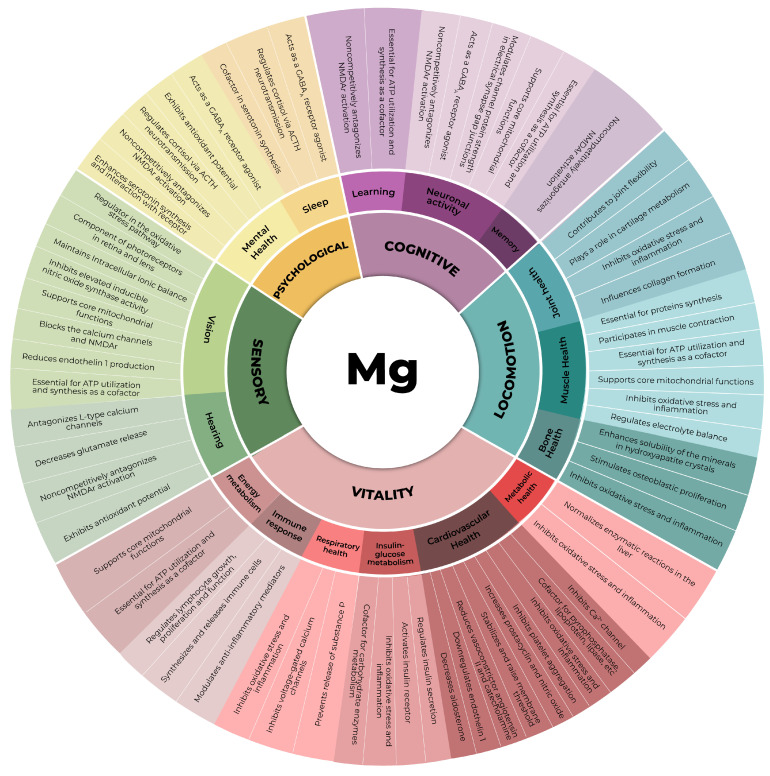
The five domains of intrinsic capacity and the subdomains in which magnesium plays a significant role [20,22,23,24,25,26,27,28]. ACTH: adrenocorticotrophic hormone, ATP: adenosine triphosphate, Ca^2+^: calcium cation, GABAA: γ-aminobutyric acid type A receptor, NMDAr: N-methyl-D-aspartate receptor.

In this context, magnesium, an essential mineral, plays pivotal roles in all domains comprising an individual’s IC (Figure 1). Despite its paramount importance and distinctive physicochemical attributes, preventing an easy replacement of its physiologic functions by any other chemical element, the aging process is notably characterized by the gradual depletion of magnesium within the organism [20,21]. This diminishment results from a confluence of factors, including the increased demand for magnesium due to its pivotal role in the maintenance of organismal homeostasis, suboptimal dietary intake, and typical age-related perturbations in absorptive processes [20,21]. In this comprehensive narrative review, the role of magnesium in preserving skeletal muscle is revisited in recent scientific literature to underscore its incomparable and irreplaceable contribution to individual mobility and overall physiological well-being during aging.

## 2. Magnesium

Magnesium (Mg) is the second most prevalent cation within human cells, following potassium (K), and is the fourth most prevalent element in the human organism. Its oxidation state is 2+. Due to its high reactivity, magnesium predominantly exists in the form of the free cation Mg^2+^ when dissolved in aqueous solutions or as an integral component of various compounds, such as carbonates, hydroxides, and chlorides, rather than in its native metallic state [20,21,29,30,31].

**Figure 2:**
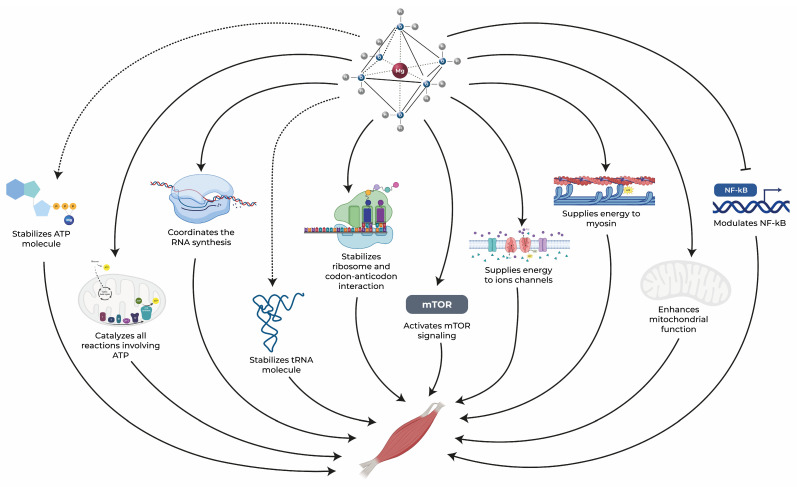
Magnesium exerts an essential role in maintaining muscle health through several pathways. Dotted arrows represent stabilization, solid arrows represent activation, and blunt-end arrows indicate inhibition. ATP: adenosine triphosphate, mTOR: mammalian target of rapamycin, NF-κB: nuclear factor kappa-B, RNA: ribonucleic acid, tRNA: transfer RNA [25,34,35,36,37,38,39,40,41].

Magnesium plays a pivotal role in almost all primary biochemical and metabolic processes within the cell, including critical functions such as oxidative phosphorylation, energy production, storage and transfer, glycolysis, and the synthesis of proteins and nucleic acids [21]. It also influences various physiological aspects in humans, including neuromuscular function, bone development, signaling pathways, lipid metabolism, and cell proliferation [20]. Magnesium is vital for maintaining genomic stability, serving as a cofactor for many DNA repair enzymes, while its deficiency may contribute to cancer development [20,25]. This remarkable element is instrumental in the mitochondrial production of adenosine triphosphate (ATP), forming Mg-ATP, and is indispensable for protein phosphorylation in cellular signaling processes [32]. Additionally, magnesium ions actively participate in the facilitation of ion transport across cell membranes, the regulation of neuron excitability, and muscle contraction. Cellular magnesium homeostasis further interconnects with the metabolism of other ions, including sodium (Na), potassium (K), and calcium (Ca), Ca^2+^-activated potassium channels, and several other mechanisms [21,33]. The involvement of a particular metal in biological processes is determined not only by its relative abundance but also by its physicochemical properties. For instance, magnesium exhibits high water solubility. In comparison to other metallic elements, magnesium presents a relatively low ionic radius but high hydration energy. Ionized magnesium frequently forms coordination complexes with six to seven water molecules. The organization of Mg and water assumes an octahedral conformation (Figure 2), and this interaction is characterized by a slower rate of water exchange than that of other metal ions [29]. Consequently, magnesium exhibits a larger size and increased stability compared to other cations, such as sodium, potassium, and even calcium [30,34]. This may elucidate the challenges encountered by magnesium in traversing narrow biological channels that are readily permeable to calcium. As a result, magnesium necessitates dehydration prior to passage through channels and transporters, a process demanding a considerable amount of energy [30].

Magnesium ions carry a 2+ positive charge, enabling them to bind with negatively charged molecules. Furthermore, magnesium exhibits a high affinity for oxygen donor ligands, including negatively charged carboxylates, phosphates, or enolate moieties. This distinctive ability to interact with diverse chemical structures positions magnesium as the most versatile intracellular cation, participating in nearly every major metabolic and biochemical process within the cell [29,30]. In general, the higher the metabolic activity of a cell, the greater its magnesium content. Additionally, due to its physicochemical characteristics, a significant portion of intracellular magnesium forms associations with ribosomes, membranes, and other charged macromolecules in the cytosol or nucleus, rendering it an essential cation for human health [30].

Table 1 illustrates the distribution of magnesium across crucial body compartments, highlighting its pivotal role in various physiological processes [20,21,30,42,43].

More than 95% of intracellular magnesium is bound to ATP, proteins, and negatively charged molecules. According to enzymatic databases, current knowledge indicates that magnesium serves as a cofactor in over 600 enzymatic reactions and may act as an activator for an additional 200 enzymes [20,30,31,45]. Consequently, magnesium assumes a fundamental role in cellular homeostasis and organ function, exerting physiological control over several vital metabolic pathways and cellular functions, including its involvement in enzyme-substrate interactions, structural functions, and membrane-related processes [20,21,30].

Magnesium homeostasis involves a dynamic balance among its intake, intestinal absorption, renal reabsorption/excretion, bone storage, and the magnesium requirements of various tissues [20,21,30]. Upon ingestion, magnesium is absorbed within the distal small intestine, and further regulatory adjustments occur within the cecum and colon. This regulation involves a passive paracellular mechanism and transcellular transport until magnesium is taken up by enterocytes [20,30]. For instance, when an individual consumes 300 mg of magnesium from their diet daily, the intestines absorb approximately 120 mg while excreting approximately 20 mg, which results in a net absorption of approximately 100 mg [30,43].

The renal excretion rate of Mg^2+^ primarily depends on serum Mg^2+^ levels. Under normal conditions, blood magnesium levels are meticulously controlled through a delicate interplay between intestinal absorption and renal excretion, even when dietary magnesium consumption is low. Consequently, the kidneys eliminate magnesium when there is an excess and reduce excretion during periods of deficiency [20,43]. Even when plasma/serum magnesium levels stay within the acceptable biochemical range, the intracellular magnesium concentrations in both bone and soft tissues (muscles and internal organs) may become depleted in cases of magnesium deficiency [20,46]. This phenomenon occurs because, in the absence of sufficient magnesium, bone and tissue stores assist in maintaining their serum concentrations by exchanging magnesium with the extracellular fluid [43,47].

In instances where magnesium intake slightly exceeds the daily requirements, excess magnesium derived from dietary sources does not pose a health risk to individuals with normal health conditions. This is attributed to the efficient elimination of excess magnesium by the kidneys through urine excretion. Consequently, hypermagnesemia is primarily observed at oral magnesium doses exceeding 2500 mg, which equates to levels surpassing ten times the established upper limit (UL). It is noteworthy that elevated magnesium levels leading to hypermagnesemia may result from the consumption of high doses through dietary supplements, pharmaceuticals, or alternative sources. Clinically, hypermagnesemia is defined by serum magnesium concentrations surpassing 1.1 mmol/L, with extreme hypermagnesemia noted when magnesium levels exceed 3.0 mmol/L [20,25,30].

Manifestations of hypermagnesemia encompass gastrointestinal symptoms such as diarrhea, nausea, vomiting, and abdominal cramping, as well as neurological symptoms like headaches, lightheadedness, or drowsiness. Notably, in skeletal muscles, lethargy, muscle weakness, paralysis, and a reduction or loss of deep tendon reflexes may ensue. Severe electrocardiographic abnormalities are also plausible, characterized by bradycardia and hypotension, potentially culminating in coma, asystole, and cardiac arrest-induced fatality [25,30,48]. These muscular and cardiac manifestations are interconnected through the complex pathophysiology of hypermagnesemia.

The cardiac and muscular symptoms associated with hypermagnesemia can be elucidated by the competition between magnesium and calcium ions for activation and deactivation sites on the type II isoform ryanodine receptor channels in cardiac myocytes. This interaction detrimentally affects cardiac contraction and relaxation. Furthermore, magnesium’s inhibitory effect on acetylcholine release in neuromuscular junctions and sympathetic ganglia may result in motor end-plate sensitivity depression, inducing arrhythmia, myocardial depression, and vasodilation. Consequently, these intricate mechanisms underscore the multifaceted nature of hypermagnesemia and its potential to instigate severe physiological consequences, encompassing both cardiac and muscular systems [49,50].

Magnesium is classified as an essential mineral. It is not synthesized by living organisms and plays a pivotal role in various biological processes and metabolic pathways. Consequently, it needs to be regularly ingested from the diet to meet recommended intake levels and prevent deficiency. In this regard, it is not only crucial to identify potential sources of magnesium but also to consider its bioavailability and factors that could influence its absorption and excretion [20,51]. Good dietary sources of magnesium include seeds, legumes (such as peas and beans), green vegetables (particularly spinach), nuts (including peanuts, cashews, almonds, and Brazil nuts), whole grain breads and cereals (such as millet and brown rice), select fruits (such as dried apricots, raisins, dried bananas, and avocado), cocoa, and seafood [20,30,51,52].

At birth, the human body has approximately 760 mg of magnesium, which increases to approximately 5 g by the age of 4–5 months [20,25,52]. The total magnesium content in the human body is estimated to be approximately 24 g (equivalent to 1 mole) [25,30]. Normal magnesium concentrations in the body typically fall within the range of 0.75 to 0.95 mmol/L (1.7–2.5 mg/dL or 1.5–1.9 mEq/L). A serum magnesium level below 0.75 mmol/L (1.8 mg/dL) is indicative of magnesium depletion, a condition known as hypomagnesemia [30,43,53]. To ensure the maintenance of consistent plasma magnesium levels, regulatory authorities responsible for dietary intake recommendations, such as the WHO, the American National Academy of Medicine (NAM), the Food and Agriculture Organization (FAO), and the European Food Safety Agency (EFSA), have established guidelines for the healthy population, as detailed in Table 2.

Despite the well-established recommended daily intake guidelines, the average dietary magnesium intake often falls short of meeting these recommendations, with levels significantly lower than the recommended daily intake [21,28]. As a result, subclinical magnesium deficiency is not uncommon in the general population. In fact, nearly two-thirds of Americans consume magnesium in quantities below the RDA, and a similar situation is prevalent in Europe and in Brazil [20,28,54,55]. Notably, although the kidneys help to regulate urinary magnesium excretion to prevent hypomagnesemia, habitual low magnesium intake or excessive losses due to various factors and conditions can eventually culminate in subclinical magnesium deficiency [20].

In addition, it is important to mention that the measurement of magnesium levels within the body remains a challenge, making the diagnosis of hypomagnesemia or hypermagnesemia complex. This difficulty arises because serum magnesium concentrations do not consistently reflect the magnesium content in various body compartments. Thus, the presence of an acceptable biochemical serum magnesium level does not necessarily eliminate the possibility of magnesium deficiency [20,56].

In fact, chronic magnesium deficiency is prevalent among the aging population, typically stemming from a reduction in both dietary magnesium intake and intestinal absorption [20,56]. In terms of dietary intake, aging often results in a diminished appetite due to factors such as compromised oral health, a decreased sense of smell and taste, impairment of vision and hearing, and anorexia linked to depression [57]. Other contributing factors include a decreased capacity to purchase and prepare food, financial constraints limiting access to adequate nutrition, shifts in energy requirements, decreased physical activity, and the development of sarcopenia, which can lead to a loss of self-sufficiency and increased social isolation [57]. All these factors collectively contribute to malnutrition in the elderly and are likely to result in diets that are deficient in essential nutrients, including magnesium [43]. Information from the National Health and Nutrition Examination Survey (NHANES) III has corroborated that aging represents an additional risk factor for insufficient magnesium consumption, with magnesium intake progressively decreasing with age [20,21].

The intestinal absorption of magnesium tends to decline with advancing age, which is a significant factor contributing to magnesium deficits in the aging population [21]. Age-related alterations in magnesium intestinal absorption may be exacerbated by changes in vitamin D homeostasis that often occur in older individuals [43,58]. Of note, magnesium and vitamin D share a reciprocal relationship. Vitamin D enhances magnesium absorption, whereas magnesium deficiency can lower vitamin D levels [20,59,60]. Magnesium is also required for the activity of key enzymes needed for the conversion of 25(OH)D to its active form, 1,25(OH)_2_D_3_, and facilitates the transfer of vitamin D to target tissues [61,62]. It is also necessary for the enzymatic inactivation of this vitamin [63]. Given that vitamin D deficiency is also highly prevalent among elderly individuals [64], the interplay between these two nutrients can further exacerbate their deficiencies in this population. Moreover, decreased kidney function and tubular reabsorption, which are frequent in elderly individuals, may further contribute to magnesium loss. Additionally, the estrogen deficiency experienced by aging women and men can exacerbate the decrease in magnesium absorption and lead to its excessive urinary excretion (hypermagnesuria) [20,21].

In addition, aging is commonly linked to magnesium deficiency due to the presence of age-related chronic diseases and the use of polypharmacotherapy, which involves the treatment of multiple medical conditions with various medications [21,43]. Pathological conditions, such as insulin resistance, type 2 diabetes, alcoholism, hyperadrenocorticism, stroke, acute myocardial infarction, HIV/AIDS, and hypertension, along with the use of multiple medications commonly prescribed to the elderly (including antivirals, antiepileptic drugs, antibiotics, antihistamines, proton pump inhibitors, antacids, and H2 blockers), contribute to magnesium deficits. Diuretics, for example, are among the most frequently prescribed drugs for hypertension treatment. Diuretic therapy can lead to excessive urinary magnesium loss, and diuretic-induced hypomagnesemia frequently coincides with hypokalemia. Consequently, the assessment of magnesium levels in patients, whether elderly or young, who present with hypokalemia is recommended [21,43].

Initial indications of magnesium deficiency often manifest as nonspecific symptoms, including weakness, muscle spasms, reduced appetite, fatigue, nausea, and vomiting. Subsequently, more pronounced symptoms may include muscle contractions and cramps, numbness, tingling, alterations in personality, and the onset of depression. In cases of severe magnesium depletion, where serum magnesium levels drop below 0.4 mmol/L, individuals may experience coronary spasms, abnormal heart rhythms, ventricular arrhythmias, tetany, and seizures [20,30]. Finally, hypomagnesemia and/or chronic magnesium deficiency can have wide-ranging impacts on nearly every organ and bodily system, potentially aggravating or contributing to pathological consequences and resulting in life-threatening complications [20,65].

Underscoring the importance of adequate magnesium intake in the aging population, preliminary data from Struijk et al. (2023) suggest that adequate magnesium intake through diet was associated with a 16% lower risk of developing frailty in elderly women [32]. In men, an increase of 100 mg in daily magnesium intake reduces the risk of frailty by 22% [39]. Hence, it becomes evident that magnesium plays a pivotal role in the overall health and well-being of individuals, especially as they age. Its deficiency, whether due to dietary inadequacies, physiological changes, or medication-induced losses, can have profound implications for an individual’s health status. The association between magnesium intake and a reduced risk of frailty underscores its importance in maintaining physical integrity. As frailty is closely linked to muscle health, it is imperative to further investigate the specific role that magnesium plays in skeletal muscle function and maintenance.

## 3. The Role of Magnesium in Muscle Health

Skeletal muscle health is fundamental to human functionality, mobility, and overall well-being [66]. During the natural process of aging, an individual’s mobility transcends being a mere measure of physical capability; it evolves into a dynamic reflection of their comprehensive health status and quality of life. The ability to move autonomously and unrestrictedly significantly influences multiple facets, including vitality, cognitive function, sensory perception, and psychological well-being. Several studies have demonstrated that consistent engagement in physical activities, which relies heavily on the integrity of skeletal muscle, is associated with enhanced cognitive performance, including improved attention, executive functions, and memory. Furthermore, physical activity triggers the release of endorphins, which are natural mood-elevating agents. As aging progresses, there is a noticeable decline in muscular function, resulting in movement restrictions, increased dependency, and, subsequently, potential negative effects on emotional and mental health [66,67,68,69,70].

Within this context, magnesium stands out as a vital element whose distinct chemical and physical properties position it as a crucial component in the regulation of nearly all biological processes within cells. In the absence of adequate magnesium levels, the entire organism is impacted, as no other chemical element can effectively assume its multifaceted roles. Notably, skeletal muscle houses approximately 20% of the body’s total magnesium [71]. This essential mineral is intricately associated with various aspects of skeletal muscle function that are negatively impacted in aging subjects. It plays a central role in processes such as protein synthesis, energy production, and muscle contraction while also offering anti-inflammatory and antioxidant benefits, as illustrated in Figure 2 [21,47,72]. In the subsequent sections, the comprehensive role of magnesium in the skeletal muscle health of elderly individuals will be explored, highlighting its significance across these physiological processes.

### 3.1. Energy Metabolism

The human body operates through intricate physiological processes that require significant energy reserves. Key tissues, especially the brain and muscles, stand out as primary energy consumers within this biological system [73,74]. In resting conditions, an average adult brain and skeletal muscle each consume approximately 250 mL O_2_/min, accounting for approximately 20% of the total O_2_ consumption for each tissue [74]. Their pivotal roles in maintaining overall bodily function dictate that a consistent and sufficient energy supply is indispensable for their optimal performance. A diminished energy provision can critically impede these essential biological processes, potentially compromising the health and efficiency of these tissues [73,74]. Given this context, it becomes imperative to elucidate the mechanisms underlying energy metabolism and to understand the contribution of magnesium in this intricate system.

As with many other systems and tissues, skeletal muscle undergoes numerous structural and functional transformations with aging, including mass loss. These changes serve as key contributors to morbidity and frailty. Age-related muscle deterioration, diminished strength, and impaired cellular energy metabolism culminate in reduced physical capability [75].

Each muscle fiber (myocyte) contains hundreds of thousands of mitochondria, which are known as the cellular “powerhouses” [76]. A significant amount of the energy needed for human physiological functions is generated by these organelles via electron movement through the respiratory chain [75]. Diminished mitochondrial function in muscles might play a role in age-related muscle dysfunction and decreased aerobic capacity [75].

Energy metabolism within cells is a complex process, with magnesium serving a crucial role, which is particularly essential for mitochondrial health and function [77]. Insufficient magnesium levels can result in decreased mitochondrial efficiency and increased production of reactive oxygen species (ROS). ROS can subsequently result in structural and functional damage to vital molecules, including DNA and proteins [77], all of which are associated with aging.

Supporting this notion, a study revealed that muscle tissue from magnesium-deficient animals exhibited mitochondrial damage, which presented as swelling and ultrastructural changes [78]. Conversely, the oxidative mitochondrial decay associated with aging might predispose individuals to hypomagnesemia. Wilson et al. (2004) indicated that a mutation in a mitochondrial gene led to decreased magnesium levels in the bloodstream. This reduction became more pronounced as the subjects aged, possibly because reabsorption of magnesium at the distal convoluted tubule of the kidney nephron demands a significant amount of ATP, which diminishes with impaired mitochondrial function [79].

Notably, magnesium in mitochondria constitutes one-third of its total cellular level, highlighting its pivotal role in cellular energy metabolism [29,72]. This essential mineral is critical in supporting core mitochondrial functions, including the electron transport chain, oxygen detoxification, and the production of ATP, which acts as the primary energy currency in cells, driving numerous physiological functions. In fact, magnesium is essential for all phosphorylation processes and reactions that entail the transfer and utilization of ATP [34,77]. Magnesium exists not only in complex with ATP but also as a component of nucleic acids and membranes [29,72].

Magnesium is needed for the activity of all rate-limiting glycolytic enzymes [47]. Moreover, the importance of magnesium in energy metabolism and muscle function is underscored by its involvement in the Mg-ATP complex, an indispensable entity for the sliding filament mechanism of myofibrillar contraction and relaxation in striated muscles [72]. Most of the ATP within cells is found as Mg-ATP complexes, representing its biologically active form [80]. Magnesium is crucial for catalyzing all reactions involving ATP, helping to maintain the polyphosphate chain of ATP in a conformation that promotes enzyme binding [20]. This importance of Mg binding to ATP arises from several roles it plays, as described below, ultimately enhancing the specificity of enzyme-substrate interactions by amplifying the binding energy [80].

Magnesium ions (Mg^2+^) counteract the negative charges on the ATP polyphosphate chain, which minimizes nonspecific ionic interactions between the enzyme and the polyphosphate group [80]. Therefore, the reactions are specific and efficient. Additionally, Mg^2+^ increases the number of interaction points between the ATP-Mg complex and enzymes, thereby enhancing binding energy and ensuring that the ATP molecule is positioned appropriately for enzymatic actions [80]. Furthermore, these magnesium-ATP interactions ensure that the nucleotide remains in well-defined structures, allowing enzymes to bind with precision. Such interactions also prime the ATP molecule by weakening its terminal O-P bond, facilitating the efficient transfer of phosphate, a foundational process in cellular energy mechanics [20].

It is worth noting that numerous prevailing theories on aging, which aim to elucidate the mechanisms behind sarcopenia, have turned their focus to the mitochondria due to their dual role in governing both life and death processes within myocytes [76]. These organelles are not just primary generators of cellular energy through Mg-ATP; they are also crucial regulators of apoptosis, which is a programmed cell death pathway [76]. As previously highlighted, aging is often associated with poor magnesium status. This can in turn subsequently influence the age-related deterioration of mitochondrial function, leading to energy depletion within the cell. Such dynamics can trigger apoptosis in aging skeletal muscles, further contributing to the age-associated loss of function [21,47,76,81].

Beyond its direct role in mitochondria, energy production, and muscle contraction, magnesium is also integral for cellular signaling processes. Specifically, the Mg-ATP complex plays a crucial role in phosphorylating proteins and in the synthesis and activation of cyclic adenosine monophosphate (cAMP). This important cell-signaling molecule participates in a plethora of biochemical processes, reinforcing the diverse and central roles of magnesium within cells [29,72,82]. Another interesting interplay is the relationship between magnesium and calcium within muscle cells. The uptake and release of calcium from sarcotubules are intimately connected to Mg-ATP levels. This association implies that even subtle changes in intracellular magnesium concentrations can profoundly influence the contractile performance of muscle cells [72].

Given that both magnesium deficiency and sarcopenia are more prevalent in the aging population and that magnesium plays a central role in muscle ATP production, a hypothesis has been posited suggesting that a compromised magnesium status might be a contributing factor to sarcopenia observed in the later stages of life [72,77]. Supporting this notion, several studies conducted in aging as well as young volunteers found that the magnesium status in the organism or its supplementation strongly affects muscle performance [77,83,84,85,86]. This is likely attributed to the pivotal role of magnesium in energetic metabolism, transmembrane transport, and muscle contraction and relaxation [77,85].

### 3.2. Protein Synthesis

Proteostasis is a term used to describe the complex and tight network responsible for maintaining protein homeostasis within cells [87], and this process plays a crucial role in regulating several physiological processes within the human body. Proteins, composed of carbon, hydrogen, oxygen, and nitrogen, serve as intricate macronutrients with multifaceted roles in the body. They contribute to structural integrity, regulate vital cellular and physiological processes, act as effectors, and, at times, serve as an energy source [88,89].

Proteostasis involves several processes that can be summarized as four main arms: protein synthesis, folding, degradation, and quality control mechanisms [90]. It is noteworthy that any perturbation in this delicate balance can have significant implications for the health of both individual cells and the organism as a whole [87,91,92,93]. The disruption of protein homeostasis is an important component among the seven fundamental determinants of the aging process and is linked to the pathogenesis of conditions such as neurodegenerative disorders, cardiovascular disease, and sarcopenia [92,94,95,96,97].

Skeletal muscle tissue is predominantly composed of proteins, as it contains 50–75% of all proteins in the body [98]. Consequently, maintaining protein homeostasis is crucial for the preservation of skeletal muscle mass. However, the aging process introduces a notable phenomenon termed anabolic resistance, in which the capability for protein synthesis is adversely affected. This anabolic blunting persists even in the presence of conventional anabolic stimuli, such as feeding and exercise, linking this impairment to prevalent conditions like sarcopenia and frailty among the elderly [93,99,100,101,102]. Using stable isotope methodologies, Wall et al. (2015) observed that postprandial muscle protein synthesis rates were 16% lower in older individuals (75 ± 1 years) than in their younger counterparts (22 ± 1 years) [103]. A reduction in the translation rate is one of the age-related changes observed in protein synthesis [93]. Furthermore, the aging process is linked to a decrease in the effectiveness of protein recycling systems, resulting in the buildup of damaged proteins and other molecules, which could hinder cell functionality and contribute to age-associated dysfunction [93].

Protein synthesis is one of the most intricate and energetically demanding anabolic processes in the cell [100,104]. This biologically vital process accounts for the consumption of a substantial portion of ATP, with over 70% of ATP reserves allocated to support various biosynthetic pathways [90]. The ribosome is a cellular organelle responsible for protein synthesis [105] that relies on amino acids as the fundamental building blocks for synthesizing proteins within all tissues of an organism [36]. In addition to ribosomes and amino acids, protein synthesis requires a diverse range of cellular components, including messenger RNA (mRNA) [36] and essential minerals [106].

The regulation of protein synthesis encompasses multiple stages of transcription and translation, and this process is crucial for the production of ribosomal RNAs and proteins that are involved in muscle contraction and metabolism [107]. Within the transcriptional phase, two magnesium cations (Mg^2+^) are localized at the active site of RNA polymerase. These cations coordinate the synthesis of RNA, playing a pivotal role in the condensation of nucleoside triphosphates (NTPs) [25,108]. One of the Mg^2+^ ions contributes to the formation of a new phosphodiester bond, while the other actively participates in stabilizing the pentacovalent transition state of the enzyme [108]. In the synthesis of ribosomal RNA (rRNA), which carries a significant negative charge, Mg^2+^ chelation serves to reduce electrostatic repulsion [35,90].

Mg^2+^ also assumes a critical role in the folding of transfer RNA (tRNA) [109]. In fact, Mg is indispensable for all RNA folding processes and energetic states due to its ability to form a rigid and tightly octahedral structure with oxygen atoms incorporating phosphate groups [109]. On the translational level, Mg^2+^ emerges as an indispensable component in stabilizing the secondary structure of ribosomes [106,109,110]. This structural stability is fundamental in all steps of translation, particularly in the initiation process of peptide bond formation [90,111].

Furthermore, Mg^2+^ plays an essential role in mediating the bonds between rRNA and ribosomal proteins (rProteins) [106,109] by activating water molecules that facilitate the recognition of rProteins [112]. Additionally, Mg^2+^ aids in stabilizing the codon-anticodon interaction at the A site and affects the binding of ribosome recycling factor (RRF) to ribosomes [106]. Notably, ribosome degradation becomes particularly important when the magnesium concentration is limited, as the recycling process releases Mg^2+^ ions for essential cellular activities [90].

Finally, the mammalian target of rapamycin (mTOR) plays a pivotal role in stimulating protein synthesis while concurrently inhibiting proteolysis [37]. Magnesium is intricately involved in this process, not only in its role in the energy system but also through its ability to activate mTOR signaling [113]. This pathway is integral for initiation, elongation, and ribosome biogenesis [114]. Given the central role of mTOR in anabolic and catabolic muscle pathways and the influence of magnesium on its activation, both mTOR and magnesium present promising targets for interventions aimed at countering age-related muscle loss [37].

As shown here, magnesium plays a pivotal role in protein synthesis, influencing both transcriptional and translational processes in skeletal muscle tissue. Its involvement in RNA synthesis, ribosomal stabilization, and activation of the mTOR signaling pathway highlights its importance in maintaining muscle health. Given the age-related decline in protein synthesis, ensuring optimal magnesium levels might be key to addressing muscle degeneration and sarcopenia in elderly individuals.

### 3.3. Anti-Inflammatory and Antioxidant Activities

Inflammation is recognized as one of the seven pillars of aging [94,115]. Notably, the chronic low-grade inflammation observed in older individuals, a condition referred to as “inflammaging”, is a pivotal risk factor for frailty, morbidity, and mortality. Additionally, it is a common feature of various age-related diseases and has been associated with numerous adverse effects on muscle health [115,116,117,118]. In fact, inflammaging has been proposed as an underlying mechanism of muscle decline and sarcopenia [119,120,121].

The inflammatory process can inhibit muscle regeneration after injury or exercise, thereby fostering muscle disuse atrophy and rendering individuals more susceptible to recurrent muscle damage [117,122,123]. Additionally, the presence of chronic inflammation is closely associated with insulin resistance. Insulin resistance is a highly prevalent condition among the elderly that diminishes intracellular glucose levels and can lead to muscle loss [112,115,124]. Moreover, inflammation can disturb the balance between muscle protein synthesis and degradation, ultimately contributing to muscle wasting [125,126,127]. In a study conducted by Merritt et al. (2013), subjects with mean ages of 61 and 76 years exhibited higher expression of several genes, including the proinflammatory cytokines tumor necrosis factor (TNF)-α and interleukin (IL)-6, as well as TNF-like weak inducer of apoptosis signaling (TWEAK), following the induction of modest muscle damage than that in individuals with a mean age of 40 years. This finding underscores the potential relevance of inflammation management as a promising strategy to mitigate age-related muscle damage [128].

The anti-inflammatory properties of magnesium have been extensively documented in the preclinical and clinical scientific literature [129,130,131,132,133]. In fact, studies have consistently shown that a low magnesium status correlates with increased low-grade systemic inflammation, as evidenced by elevated levels of proinflammatory markers such as TNF-α, IL-1β, and C-reactive protein (CRP) and a reduction in the levels of anti-inflammatory cytokines [72,134]. It also impacts immune responses by activating leukocytes and macrophages, causing endothelial dysfunction and inflammatory syndrome [134,135]. Additionally, magnesium deficiency can impact mast cells by affecting their histamine secretion, and histamine is a key component in inflammatory responses [71]. Conversely, magnesium repletion therapy induces an anti-inflammatory response and reduces proinflammatory marker levels in rats initially deficient in magnesium [136,137].

Recent research has focused on elucidating the pathway linking sustained chronic inflammation to oxidative stress, a process implicated in numerous chronic diseases, including sarcopenia [71,72,118]. The interplay of inflammation and oxidative stress can influence multiple intracellular signaling pathways, disrupting mitochondrial function and the equilibrium between protein synthesis and degradation. This prompts apoptosis and ultimately results in the loss of muscle mass [72,118]. Moreover, oxidative stress can trigger several transcription factors, including NF-κB, AP-1, and NRF2, which, when activated, modulate the expression of more than 500 genes, including inflammatory cytokines, chemokines, and molecules integral to oxidative stress defense. Thus, there is a profound interconnection between oxidative stress and chronic inflammation, and both of these processes play key roles in age-related muscle atrophy [71,118,138].

Previous studies on inflammation, including clinical studies and those based on animal and cellular models, have consistently shown a link between low magnesium status and the onset of oxidative stress and compromised antioxidant defense systems [40,72,139]. Several studies have indicated that magnesium deficiency is characterized by reduced antioxidant defenses and elevated levels of oxidative stress markers, including those related to lipid, protein, and DNA oxidative modifications, resulting in enhanced free-radical-induced tissue damage [72,139,140,141,142,143,144,145,146].

For instance, Boparai et al. (2007) discovered protein and lipid oxidation in the liver and plasma of rats subjected to a magnesium-deficient diet [142]. In the study by Gueux et al. (1995), lipoproteins (VLDL and LDL) from magnesium-deficient rats showed increased susceptibility to CuSO_4_-induced oxidative damage compared to control rats. Additionally, tissues exposed to lipid peroxidation induced by iron (Fe) exhibited higher levels of thiobarbituric acid-reactive substances, indicative of increased oxidative stress [140]. Accordingly, rats fed a magnesium-deficient diet showed a notable reduction in both erythrocyte and plasma magnesium levels, accompanied by a significant increase in the plasma oxidative marker malondialdehyde (MDA) and a corresponding reduction in radical-trapping antioxidant markers [141]. Reductions in the activities and levels of important molecules and enzymes related to antioxidant defenses, including glutathione (GSH), glutathione reductase (GR), superoxide dismutase (SOD), catalase, glutathione S-transferase (GST), and vitamin E, were also observed in rodents after magnesium deprivation, which further led to an increase in oxidative stress [147,148,149,150]. Conversely, magnesium supplementation has the potential to mitigate oxidative stress. In a rat model of diabetes, low magnesium levels and increased urinary excretion were correlated with increased plasma MDA and reduced hepatic expression of SOD and GST, all of which were rectified with magnesium supplementation [151].

Regarding the clinical context, studies in humans have also corroborated the interplay among low magnesium status, low-grade systemic inflammation, and oxidative stress. Song et al. (2005) found an inverse association between plasma CRP concentrations and dietary magnesium content in a cohort comprising more than 11,000 women aged 45 and older participating in the Women’s Health Study [152]. Moreover, several meta-analyses have concluded that magnesium reduces CRP levels [130,134,153]. For example, more recently, Veronese et al. (2022) conducted a comprehensive meta-analysis including 17 randomized controlled trials involving 889 participants (mean age: 46 years), and their findings revealed a significant reduction in various inflammatory markers, particularly CRP, associated with magnesium supplementation [134]. Accordingly, in human subjects chronically exposed to stress, an inverse association was observed between magnesium levels and oxidative stress markers, specifically MDA and plasma superoxide anions [154]. Additional clinical research indicates an inverse relationship between serum magnesium levels and markers of oxidative stress and inflammation [155,156]. Last, in three recent randomized controlled trials, magnesium cosupplementation with zinc, melatonin, or zinc-calcium-vitamin D was shown to reduce inflammatory and oxidative stress markers in women with polycystic ovary syndrome [157,158,159].

While the precise pathophysiological mechanisms underlying the anti-inflammatory and antioxidant effects of magnesium remain to be fully elucidated, many studies highlight its pivotal role in mitochondrial function (see Section 3.1). Magnesium supplementation has been demonstrated to enhance mitochondrial function by inhibiting mitochondrial ROS, modulating permeability, and regulating the opening of the mitochondrial transition pore [160]. Moreover, several studies have proposed that magnesium deficiency can contribute to the onset and persistence of oxidative stress and inflammation, primarily through mechanisms related to mitochondrial dysfunction [71,139,161]. Magnesium deficiency can contribute to disrupted mitochondrial functioning by promoting the uncoupling of oxidative phosphorylation, leading to electron loss in the electron transport chain, which in turn amplifies intracellular reactive species production and consequent oxidative stress [139,162,163,164]. Furthermore, reduced magnesium concentrations cause calcium accumulation in the cytosol [163,164]. This not only contributes to the uncoupling of oxidative phosphorylation but also stimulates other peroxidation pathways [139,165,166]. The overproduction of peroxynitrite induced by magnesium deficiency further intensifies mitochondrial dysfunction [167,168]. Notably, proinflammatory mediators, also induced by magnesium deficiency [72,134], can further impact mitochondrial function, thereby amplifying mitochondrial oxidative stress and perpetuating an oxidative-inflammatory cycle [41].

At the molecular level, a key mechanism of action of magnesium involves the modulation of nuclear factor kappa-B (NF-κB), which serves as a pivotal transcription factor responsible for modulating the expression of a myriad of genes. When activated by various stimuli, including Toll-like receptors (TLRs), NF-κB translocates to the nucleus, where it upregulates the expression of genes associated with inflammatory and oxidative stress responses [169]. However, its excessive activation can lead to oxidative stress and chronic inflammation [169], a hallmark of inflammaging [119,120]. In this context, several studies have shown that magnesium can effectively reduce cytokine production via the downregulation of the TLR/NF-κB signaling pathway [71,170].

In fact, preclinical data have shown that inflammation in skeletal muscle is often marked by the activation of the NF-κB signaling pathway [171]. Persistent activation of this pathway has been shown to induce significant atrophy in mouse muscle [172]; it is also activated by muscle immobilization [173]. In line with this, short-term muscle fiber-specific overexpression of either the NF-κB p65 subunit or its activating enzyme, inhibitor κB kinase 2 (IKK2), results in muscle atrophy [174]. Conversely, targeted elimination of IKK2 and the resulting decrease in NF-κB activation have been associated with enhanced skeletal muscle strength, preserved mass, and improved regeneration [175]. Hence, it is hypothesized that magnesium could attenuate age-related muscle deterioration via NF-κB modulation.

In summary, the multifaceted roles of magnesium in combating inflammation and oxidative stress underscore its potential as a therapeutic agent for age-related muscle decline. The intricate interplay among magnesium status, inflammation, oxidative stress, and mitochondrial dysfunction highlights the importance of maintaining optimal magnesium levels, especially in aging populations. While the precise mechanisms through which magnesium exerts its protective effects remain a topic of ongoing research, the current evidence underscores its potential in modulating key pathways, such as the NF-κB signaling pathway, which is pivotal in muscle health.

### 3.4. Muscle Contraction and the Equilibrium of Electrolytes

Within the muscular system, muscle contraction, defined as the activation of muscle fibers leading to their subsequent shortening, is a fundamental physiological process [69]. Skeletal muscles, in particular, are pivotal for imparting stability and strength to the entire spectrum of bodily movements [69].

Muscle strength is governed by various multifaceted factors and is closely associated with the intrinsic quality of muscle and its aptitude for contractile action, particularly among elderly individuals. Notably, the pace of strength decline resulting from the aging process exceeds the rate of muscle mass reduction [176]. As individuals age, there is a discernible reduction in the dimensions and contractile efficacy of muscle fibers [70]. This culminates in a decline in muscle strength and power and an increase in frailty, ultimately impeding functionality and postural stability or even causing immobility [66,69].

These physiological transformations can increase the risk of severe injuries, limit participation in recreational activities, and ultimately impair the ability to carry out everyday tasks, thereby compromising individual independence and overall quality of life [66,69]. Nutrition can serve as a valuable ally in counteracting muscle loss, and magnesium plays a pivotal role in this context [70].

The cycle of muscle contraction fundamentally hinges on the supply of energy, primarily achieved through ATP hydrolysis. This process is initiated by the release of calcium ions (Ca^2+^) stored within the sarcoplasmic reticulum upon stimulation from the central nervous system [66,177]. Upon release, Ca^2+^ binds to troponin C and myosin, leading to conformational alterations in these proteins and subsequently precipitating muscle contraction [25,177,178].

Magnesium is an antagonist to calcium that competes for the same Ca^2+^-binding sites and thereby exerts regulatory control over the muscle contraction process [25,178]. In the quiescent state, magnesium is present in muscle cells in concentrations approximately 10,000 times higher than calcium, effectively occupying all available Ca^2+^ binding sites. It is only upon the release of Ca^2+^ from the sarcoplasmic reticulum that magnesium is displaced. However, under conditions of magnesium deficiency, even minimal amounts of calcium can displace magnesium. This results in hypercontractility, marked by muscle cramps and spasms [25], which are common events in advanced age [179].

Furthermore, following muscle contraction, the reuptake of calcium by the Ca^2+^-ATPase of the sarcoplasmic reticulum is contingent on the presence of magnesium [180]. This process is energy intensive, requiring one ATP molecule to transport just two Ca^2+^ ions [178], and magnesium plays an essential role in stabilizing and activating cellular ATP molecules [181,182,183]. In cases of inadequate ATP reserves, muscle fibers remain in a contracted state, preventing the release of actin and myosin chains and consequently leading to muscle cramps [179]. Another clinical consequence of magnesium deficiency is the development of cardiac arrhythmias, stemming from the disruption of cardiac muscle contraction regulation [38].

The maintenance of proper electrolyte balance is integral to muscle contraction functionality, as it serves to stabilize and uphold membrane potential [184,185,186,187,188]. This, in turn, regulates the flow of ions, fluids, and other molecules within the aqueous milieu of muscle [185] while preventing the onset of muscle fatigue [189]. Magnesium ions play a pivotal role in maintaining the electrolyte equilibrium of calcium, potassium, and sodium within skeletal muscle cells [178,187]. Last, magnesium facilitates the energization of ion channels, thus supporting their proper functioning [182]. Thus, the multifaceted role of magnesium in muscle contraction, from energy provision to electrolyte balance, underscores its indispensable nature in ensuring optimal muscle function, highlighting the need for adequate magnesium levels, especially as individuals age.

### 3.5. Magnesium and Muscle Health: Evidence from Human Studies

The examination of oral magnesium supplementation’s impact on muscle-related outcomes has been the focus of numerous interventional clinical studies, detailed in Table 3. This section reviews key findings, emphasizing study design, magnesium supplementation specifics, and implications for muscle health.

Out of 26 studies in Table 3, 19 were randomized placebo-controlled trials [83,86,190,191,192,193,194,195,196,197,198,199,200,201,202,203,204,205,206] and three were randomized controlled trials without a placebo group [207,208,209]. The remaining studies had varying designs [132,210,211,212]. Dosage and duration of magnesium supplementation varied significantly across studies, ranging from 10 days to 32 weeks, and various magnesium sources were utilized, including Mg oxide, Mg sulfate, Mg citrate, Mg lactate, and chelated forms like Mg creatine chelate and Mg bisglycinate chelate.

Despite methodological limitations, such as small sample sizes, collective findings generally highlight magnesium’s positive impact on muscle health, attributed to its performance-enhancing, analgesic, and anti-inflammatory properties.

A substantial portion of the research, involving 15 studies [83,86,132,191,192,194,195,196,197,199,207,208,209,211,212] focused on the effects of magnesium on muscle-related physical performance in healthy individuals, with participants spanning untrained subjects to elite athletes. Of these studies, 10 [83,86,132,134,192,194,195,197,199,211] reported favorable outcomes with magnesium supplementation, enhancing muscle power, torque, exercise performance, lean body mass, handgrip strength, and reducing muscle soreness and markers of muscle damage. Although not directly linked to muscle health, improvements in metabolic response [199] and reductions in blood pressure [194,207] were also noted.

Studies also explored magnesium’s role in clinical populations, including patients with alcoholic liver disease [190], chronic alcoholism [193], and chronic or acute musculoskeletal low back pain [205,210]. While benefits in muscle strength were not observed in patients with alcoholic liver disease [190], improvements in muscle strength were noted in chronic alcoholics [193]. In cases of low back pain, magnesium supplementation demonstrated an analgesic effect [205,210].

The efficacy of magnesium in managing nocturnal leg cramps (NLC) yielded mixed results. Among the six studies [200,201,202,203,204,206] focusing on NLC, three specifically addressed pregnant women [200,203,204], a group particularly prone to NLC. Findings were divergent: three studies found no magnesium effect on NLC [201,203,206], whereas the others [200,202,204] observed decreases in frequency, intensity, and subjective discomfort. Factors influencing efficacy were discussed, including high baseline magnesium levels and dietary intake, potentially low magnesium supplementation dosages, the chemical form of magnesium administered, and the supplementation duration [191,194,195].

Among the studies listed, while several investigations included older participants, it is noteworthy that only one study specifically targeted the aging population [208] and none focused on the broader aspects of muscle health in this demographic, emphasizing the need for more targeted clinical research in this area. Such research is essential to establish evidence-based guidelines for magnesium supplementation to support muscle function and overall health and quality of life in the aging population.

In conclusion, while there is promising evidence supporting magnesium supplementation’s role in muscle health, further well-designed randomized controlled trials are necessary to conclusively establish its therapeutic potential in diverse muscle-related conditions, especially in the elderly.

**Table 3:** Clinical studies that have evaluated the effects of oral magnesium supplementation on muscle-related outcomes.

Author/Year	Study Design	Subjects (Age)	Intervention Dose and Duration	Source	Main Findings
**(i) Magnesium supplementation in exercise performance**					
Brilla et al., 2003 [86]	Randomized, double-blind, placebo-controlled trial	35 recreationally active healthy subjects (19–24 y)	Placebo or 800 mg of Mg and 5 g of creatine per day for 2 weeks	MgO + creatine (MgOC) or Mg creatine chelate (MgCC)	MgOC and MgCC showed increases in bw and power. Only MgCC decreased ECW and increased ICW and peak T, suggesting that MgCC affects cellular fluid compartments and may enhance muscle creatine uptake, cellular hydration, and potentially protein synthesis.
Brilla and Haley, 1992 [83]	Randomized, double-blind, placebo-controlled trial	26 untrained subjects (18–30 y)	Mg supplement to achieve an intake of 8 mg/kg bw for 7 weeks	MgO	Significant increase in strength for the Mg group vs. control group, with improvements in absolute quadriceps T, relative T adjusted for bw, and relative T adjusted for LBM.
Finstad et al., 2001 [191]	Randomized, double-blind, placebo-controlled, crossover trial	121 physically active women (21 ± 3 y)	Placebo or 212 mg of Mg for 4 weeks, followed by a 6-week washout period and treatment crossover	MgO	Mg increased resting ionic Mg levels but did not significantly affect performance or recovery indices.
Golf et al., 1998 [192]	Randomized, double-blind, placebo-controlled trial	23 competitive triathletes (29.4 ± 3.3 y)	Placebo or 17 mmol/d of Mg for 4 weeks	Mg orotate	Mg improved performance times in triathlon events, increased serum glucose and higher oxygen uptake, reduced stress responses (lower cortisol levels and leukocyte count), and showed a milder increase in CK post-test, indicating enhanced metabolic efficiency and reduced physiological stress during competition.
Kass and Poeira, 2015 [194]	Randomized, double-blind, placebo-controlled, crossover trial	13 normotensive male and female subjects (38.5 ± 5.3 y)	300 mg of Mg for 1 week (acute) or 4 weeks (chronic)	Mg citrate	Acute Mg increased bench press performance by 17.7% on day 1, with sustained performance on day 2. Chronic Mg resulted in a 32.1% performance decline on day 2. Both acute and chronic Mg reduced post-exercise SBP on day 2, while only acute Mg reduced DBP. TPR decreased with acute Mg but not with chronic Mg.
Kass et al., 2013 [207]	Randomized, controlled, pilot trial	16 healthy and physically active male subjects (19–24 y)	No treatment or 300 mg of Mg * for 14 days	MgO	Mg reduced resting and post-exercise BP, with a greater effect on resting BP in low-dietary Mg intake individuals and a more pronounced reduction in post-exercise BP in high-dietary Mg intake individuals. No change in performance indicators.
Martinez et al., 2017 [211]	Nonrandomized controlled trial	12 elite (25.3 ± 4.4 y) and 12 recreational (22 ± 3.8 y) male basketball players	No treatment or 400 mg/day of Mg for 32 weeks (measurements in 4 time points, each 8 weeks apart: T1, T2, T3, and T4)	Mg lactate	Serum Mg significantly decreased in T3 but increased after supplementation with T4. Muscle damage markers remained stable, except for creatinine, which decreased post-T2 and then increased in T3 and T4 compared to T2. Mg may prevent tissue damage associated with intense physical activity.
Zajac et al., 2020 [199]	Randomized, placebo-controlled trial	16 elite soccer players (25.6 ± 3.7)	Placebo or 500 mg of Mg (0.07 g/kg bw) for 16 weeks	Mg creatine chelate	Mg improved total time and power in the RAST, enhancing the first and sixth 35 m sprints, with no significant changes in placebo. Mg increased creatinine, lactate, and bicarbonate levels while lowering pH values post-RAST, indicating improved sprint performance and metabolic response.
Moslehi et al., 2013 [195]	Randomized, double-blind, placebo-controlled trial	74 healthy overweight women with BMI 25–30 kg/m² (40–55 y)	Placebo or 250 mg of Mg for 8 weeks	MgO	Mg increased LBM, decreased fat mass, and improved handgrip strength and TGUG vs. baseline. No significant enhancement in knee extension strength.
Setaro et al., 2014 [197]	Randomized, double-blind, placebo-controlled trial	25 professional male volleyball players (Mg: 17.42 ± 1.56 y; C: 17.85 ± 0.99 y)	Placebo or 350 mg of Mg for 4 weeks	MgO	Mg led to decreased lactate production and enhanced plyometric performance, indicating improved alactic anaerobic metabolism. Mg erythrocyte levels, [Mg]U, CK activity, and VO2 max remained within normal ranges.
Steward et al., 2019 [197]	Counterbalanced, double-blind, placebo-controlled, crossover study	9 male recreational runners (27 ± 4 y)	Placebo or 500 mg/day of Mg for 7 days before a 10 km downhill running time trial	MgO + Mg stearate	Mg lowered IL-6 levels, reduced muscle soreness, and improved the recovery of blood glucose post-exercise. No differences in glucose and lactate during the trial or in post-measures of creatine kinase or maximal muscle force.
Terblanche et al., 1992 [212]	Double-blind, placebo-controlled trial	20 marathon runners (25–49 y)	Placebo or 365 mg of Mg for 4 weeks before and 6 weeks after a marathon	Mg-L-aspartate-HCl	In Mg-replete subjects, Mg did not increase muscle or serum Mg concentrations and had no effect on marathon running performance. It also did not influence the extent of muscle damage or the rate of recovery of muscle function post-marathon.
Veronese et al., 2014 [208]	Randomized, controlled trial	139 healthy elderly women (71.5 ± 5.2 y)	No treatment or 300 mg of Mg per day for 12 weeks	MgO	Mg group showed significant improvement in total SPPB score, chair stand times, and 4-m walking speeds vs. control group. No significant differences in secondary outcomes and no serious adverse effects reported.
Selsby et al., 2004 [196]	Randomized, double-blind, placebo-controlled trial	31 weight-trained male subjects (18–24 y)	placebo or 2.5 g of Cr or Mg-creatine chelate (providing 2.5 g of Cr) for 10 days	Mg creatine chelate (MgCC)	Cr and MgCC showed increases in work performed at 70% of 1RM for the bench press vs. placebo. No significant differences between the Cr and MgCC groups in performance tests, suggesting that a low dose of Cr, with or without Mg chelation, can enhance performance.
Zorbas et al., 2010 [209]	Randomized, controlled trial	40 physically healthy male subjects (21.5 ± 3.0 y)	Control subjects (UCS); hypokinetic subjects (UES); control subject + 3.0 mmol of Mg/kg (SCS); hypokinetic subjects + 3.0 mmol of Mg/kg (SES)	MgCl_2_	Decreased muscle Mg and increased plasma Mg and urinary/fecal Mg loss in SES and UES groups vs. controls; more pronounced effects in SES. No changes in control groups.
**(ii) Magnesium supplementation in clinical conditions**					
Aagaard et al., 2005 [190]	Ranadomized, placebo-controlled trial	59 patients with alcoholic liver disease (34–61 y)	2 days of infused Mg (30 mmol in 1 L of glucose solution 55 g/L) + 6–7 weeks 12.5 mmol of Mg orally or placebo	MgSO_4_ (iv) and MgO (oral)	Muscle Mg concentration was 7% higher in the Mg-treated vs. placebo group. Mg had no effect on muscle strength or mass.
Gullestad et al., 1992 [193]	Randomized, double-blinded, placebo-controlled trial	49 chronic alcoholics (28–84 y)	Placebo or 15 mmol Mg for 6 weeks	Mg citrate lactate	Mg significantly reduced liver enzymes (ASAT, ALAT, GOT), slightly increased serum K and Mg, and significantly improved muscle strength. Alcohol consumption remained constant, indicating that the effects were due to Mg supplementation.
Bayram et al., 2021 [210]	Prospective, randomized, open-label trial	209 subjects with acute musculoskeletal low back pain (18–65 y)	NSAID + 365 mg of Mg, NSAID + paracetamol, NSAID	NK	Improvements in functional outcome and musculoskeletal pain intensity from the initial visit to the 4th day with Mg showed greater enhancement than with NSAID alone and NSAID + paracetamol. No significant difference in these improvements or lumbar mobility between groups by the 10th day.
Yousef and Al-deeb, 2013 [205]	Randomized, double-blinded, placebo-controlled trial	80 patients with chronic low back pain (56.4 ± 13.6 y)	Placebo for 6 weeks or iv Mg for 2 weeks followed by oral Mg for 4 weeks.	MgSO_4_ (iv) and MgO + Mg gluconate (oral)	Significant reduction in pain intensity (7.5 to 4.7) and improvement in lumbar spine flexion, extension, and lateral flexion movements over 6 months in the Mg group.
**(iii) Magnesium supplementation in nocturnal leg cramps**					
Maor et al., 2017 [206]	Randomized, double-blind, placebo-controlled trial	88 male and female subjects with NLC (64.9 ± 11.1 y)	Placebo or 520 mg of Mg for 4 weeks	MgO	There was a mean change in weekly NLC of −3.41 in Mg group and −3.03 in placebo group, with no significant difference between groups. No differences were observed in severity and duration of NLC, QoL, or QoS.
Dahle et al., 1995 [200]	Randomized, double-blind, placebo-controlled trial	73 pregnant women with NLC (NK age)	Placebo or 360 mg of Mg for 3 weeks	Mg citrate lactate	Mg decreased NLC vs. baseline and placebo, but did not significantly increase serum Mg levels, excess magnesium being excreted as measured by an increase [Mg]U.
Frusso et al., 1999 [201]	Randomized, double-blind, placebo-controlled, crossover trial	45 male and female subjects with NLC (28–87 y)	Placebo or 1800 mg of Mg for 30 days, followed by a 30-day washout period	Mg citrate	No significant differences between Mg and placebo in any of the evaluated outcomes, including mean number of cramps.
Roffe et al., 2002 [202]	Randomized, double-blind, placebo-controlled, crossover trial	73 male and female subjects with NLC (Mg: 61 ± 11 y, C: 64 ± 10 y)	Placebo or 300 mg of Mg for 6 weeks	Mg citrate	There was a trend towards less NLC on Mg (*p* = 0.07). No difference in cramp severity and duration between groups. Significantly more subjects thought that the treatment had helped after Mg than after placebo, suggesting that Mg may be effective in NLC.
Nygaard et al., 2008 [203]	Randomized, double-blind, placebo-controlled trial	45 pregnant women with NLC (Mg: 32 ± 4 y, C: 30 ± 3 y)	Placebo or 360 mg of Mg for 2 weeks	Mg citrate lactate	Mg had no significant effect on frequency or intensity of NLC.
Supakatisant and Phupong, 2012 [204]	Randomized, double-blind, placebo-controlled trial	86 pregnant women with NLC (Mg: 29 ± 6 y, C: 29 ± 5 y)	Placebo or 300 mg of Mg for 4 weeks	Mg bisglycinate chelate	Mg resulted in significant lower cramp frequency and intensity vs. placebo. No significant differences in terms of side effects.
**(iv) Magnesium supplementation and its distribution**					
Wary et al., 1999 [198]	Randomized, double-blind, placebo-controlled trial	30 young healthy male volunteers (23.7 ± 4.5 y)	Placebo or 12 mmol Mg for 1 month	Mg lactate	Significant change in 24-h [Mg]U after Mg treatment. No differences in other clinical, biological, or Mg status parameters between groups, including intracellular free Mg concentrations of skeletal muscle.

1RM, 1 repetition maximum; ALAT, alanine aminotransferase; ASAT, aspartate aminotransferase; BMI, body mass index; bw, body weight; C, control; CK, creatine kinase; Cr, creatine; DBP, diastolic blood pressure; ECW, extracellular water; GOT, glutamic oxaloacetic transaminase; ICW, intracellular water; iv, intravenously; LBM, lean body mass; NK, not known; NLC, nocturnal leg cramp; NSAID, nonsteroidal anti-Inflammatory drug; QoL, quality of Life; QoS, quality of sleep; RAST, repeated sprint ability test; SBP, systolic blood pressure; SCS, supplemented control subject; SES, supplemented experimental subject; UCS, unsupplemented control subject; UES, unsupplemented experimental subject; SPPB, short physical performance battery; T, torque; TGUG, time get up and go test; TPR, total peripheral resistance; VO2max, maximum oxygen volume; y, years; [Mg]U, urinary magnesium concentration. * 300 mg refers to the weight of magnesium oxide (dosage of elemental magnesium not provided).

## 4. Conclusions

In summary, the intricate relationship between magnesium and skeletal muscle function, especially in the context of aging, is of paramount importance. As the aging process unfolds, the progressive physiological and structural changes in muscles contribute to disability and frailty among elderly individuals. Magnesium plays multifaceted roles in muscle function, including its roles in contraction, electrolyte balance, energy provision, and anti-inflammatory and antioxidant defense, and has emerged as a critical mineral in preserving muscle health and functionality.

A combination of factors, including low magnesium intake, reduced gastrointestinal absorption, and increased renal excretion, contribute to the common occurrence of chronic magnesium deficiency among aging populations. Hence, ensuring an adequate magnesium status becomes paramount, particularly for elderly individuals, to mitigate muscle-related complications and to promote overall quality of life. The cost-effectiveness of magnesium supplementation advocates for its consideration in geriatric health care. Further studies are warranted to address the broader implications of magnesium for muscle health.

## Figures and Tables

**Table 1:** Magnesium Distribution in the Human Body [20,21,30,42,43,44].

Location	Magnesium Distribution
Bone	50–60% of total magnesium (24–29 g), with approximately one-third being exchangeable
Soft Tissues (muscles and other organs)	34–39% of total magnesium
Blood	Less than 1% of the body’s magnesium
Plasma	60% ionized, 30% bound to albumin, 10% complexed with serum anions (phosphate and citrate)

**Table 2:** Magnesium intake recommendations.

Life Stage	PRI (mg)	AR (mg)	UL * (mg)	RDA-DRI (mg)	DRV-AI (mg)
Birth to 6 months	-	-	Nd	30	-
Infants 7–12 months	80	Nd	Nd	75	80
Children 1–3 years	80	65	250	80	170
Children 4–6 years	100	85	250	130	230
Children 7–10 years	150	130	250	240	230
Teen boys 11–18 years	240	170–200	250	410	300
Teen girls 11–18 years	240	170–200	250	360	250
Men	240	170	250	400–420	350
Women	240	170	250	310–320	300
Pregnant	240	170	250	350–400	300
Breastfeeding	240	170	250	310–360	300

Population reference intake (PRI), average requirement (AR), recommended dietary allowance (RDA), dietary reference intake (DRI), dietary reference value (DRV), adequate intake (AI), and tolerable upper intake level (UL). * The UL value refers to the intake of magnesium through pharmaceutical or supplements, in addition to the magnesium already present in the diet. Adapted from a previous study [20].

## Data Availability

Not applicable.
